# The prevalence and molecular characterization of *Porcine teschoviruses* in Guangdong Province, China

**DOI:** 10.1128/spectrum.04214-25

**Published:** 2026-09-09

**Authors:** Xingyao Feng, Zirui Tang, Mingjie Liu, Shengnan Chen, Feng Wen, Kaiyin Hu, Lisha Chen, Rui Zhou, Jinyue Guo

**Affiliations:** 1 College of Animal Science and Technology, Foshan University 47868, Foshan, Guangdong, China; 2 Guangdong Findergene Biotechnology Co Ltd., Foshan, Guangdong, China; Public Health Agency of Canada, Winnipeg, Manitoba, Canada

**Keywords:** *Porcine teschovirus*, molecular characterization, prevalence, Guangdong Province

## Abstract

*Porcine teschoviruses* (PTVs) are globally endemic and widely circulate within pig populations. This study aimed to investigate the prevalence and genetic characteristics of PTVs in Guangdong Province, China. A total of 341 fecal and 99 tissue mixture samples were collected from pigs in seven cities across the province. These samples were screened for PTVs using reverse transcription-polymerase chain reaction (RT-PCR). The overall PTV positivity rate was determined to be 19.32%. In this study, the PTV3 PTV/CN/GD/316 strain was successfully isolated from a tissue mixture sample obtained from a pig exhibiting diarrhea. The isolation was performed using swine testicular (ST) cells. The complete viral genome of this isolate measured 6,999 nucleotides (nt) and encoded a polyprotein of 2,205 amino acids. Phylogenetic analysis of the complete VP1 gene identified seven distinct PTV genotypes (PTV2, PTV3, PTV4, PTV9, PTV14, PTV17, and PTV19) among the 21 PTV isolates obtained, with PTV3 being the dominant genotype (38.10%), followed by PTV9 (19.05%). Furthermore, our findings revealed a significant co-infection of PTVs with other common swine pathogens, including PCV2, PEDV, PCV3, PRRSV, CSFV, and PDCoV. The co-infection rate with these viruses was remarkably high, reaching 97.65%. Collectively, these data demonstrate the widespread circulation of PTVs in pig farms in Guangdong Province and highlight the prevalence of co-infection scenarios with other viral agents. The genetic analyses of all available PTV VP1 sequences also underscore the considerable diversity of PTV genotypes circulating within the Guangdong region.

**IMPORTANCE:**

Currently, PTVs are widespread globally, with their infection rates showing a rising trend. The prevalence and molecular characterization of PTVs help prevent and control the spread of diseases. The findings of this study indicate a high prevalence and considerable genetic diversity of PTVs in certain areas of Guangdong Province, China. The occurrence of co-infection with other pathogens is also relatively common. Based on these data, we can better safeguard swine health and mitigate the economic impact of these infections on the agricultural industry.

## INTRODUCTION

*Porcine teschoviruses* (PTVs), belonging to the genus Teschovirus within the family Picornaviridae, are non-enveloped viruses characterized by a single-stranded, positive-sense RNA genome (1). According to the International Committee on Taxonomy of Viruses (ICTV), PTVs encompass 13 known serotypes, and at least 19 subclades of Teschovirus A and 3 subclades of Teschovirus B have been identified (2, 3). The PTV genome is approximately 7.1 kb in length, featuring a single long open reading frame (ORF) that encodes both structural proteins (L, VP4, VP2, VP3, and VP1) and nonstructural proteins (2A–C and 3A–D) through proteolytic processing (4). VP1, a major component of the viral capsid, is also the most genetically diverse protein within the PTVs (5). Consequently, phylogenetic analysis of the VP1 gene has become a reliable method for characterizing PTV genotypes (6).

PTVs are widely endemic in pig herds and are commonly isolated from fecal samples of both domestic pigs and wild boars (7). The virus was first reported in the town of Teschen, Czech Republic, in the early 20th century, where it caused severe central nervous system disorders in pigs (8). Since then, PTVs have spread globally, with documented occurrences in Europe (Spain, Hungary, and the Netherlands), South America (Brazil), North America (Canada, the United States, and Haiti), and Asia (Japan, India, and China) (9–18). However, most PTV cases reported outside of the initial Teschen outbreak have exhibited moderate neurological symptoms and low mortality rates. Typically, severe PTV1-associated neurological disease is referred to as Teschen disease, whereas milder forms of PTV-associated illness are known as Talfan disease. Notably, the most recent severe outbreak of PTV1, characterized by high mortality, occurred in the Republic of Haiti in 2009 (15). In China, the first documented PTV isolate (Swine/CH/IMH/03) was reported in 2003, and subsequent epidemiological studies on PTV in the country have been published since then (19). Due to PTV’s environmental persistence, there is an increased probability of both single and co-infection with other pathogens, including porcine pseudorabies virus (PRV), porcine reproductive and respiratory syndrome virus (PRRSV), transmissible gastroenteritis virus (TGEV), porcine circovirus type 2 (PCV2), and classical swine fever virus (CSFV) (20–23). Co-infection of PTVs with other pathogens may exacerbate clinical signs, complicate disease management, and pose significant challenges to prevention and control efforts (24).

Building upon the insights gained from prevalence monitoring, this study reports the successful isolation and characterization of the PTV3 PTV/CN/GD/316 strain. Following isolation, approximately complete genome sequences of this strain were obtained and subjected to rigorous comparative analysis against a comprehensive data set of known PTV sequences available in GenBank. To elucidate the evolutionary trajectory and genetic relationships of this isolate within the broader PTV landscape, detailed phylogenetic and genetic divergence analyses were performed. These analyses aimed not only to classify the isolate’s precise genotype and serotype affiliations but also to identify potential genetic markers associated with its specific characteristics or origin.

## MATERIALS AND METHODS

### Samples collection

**Fig 1:**
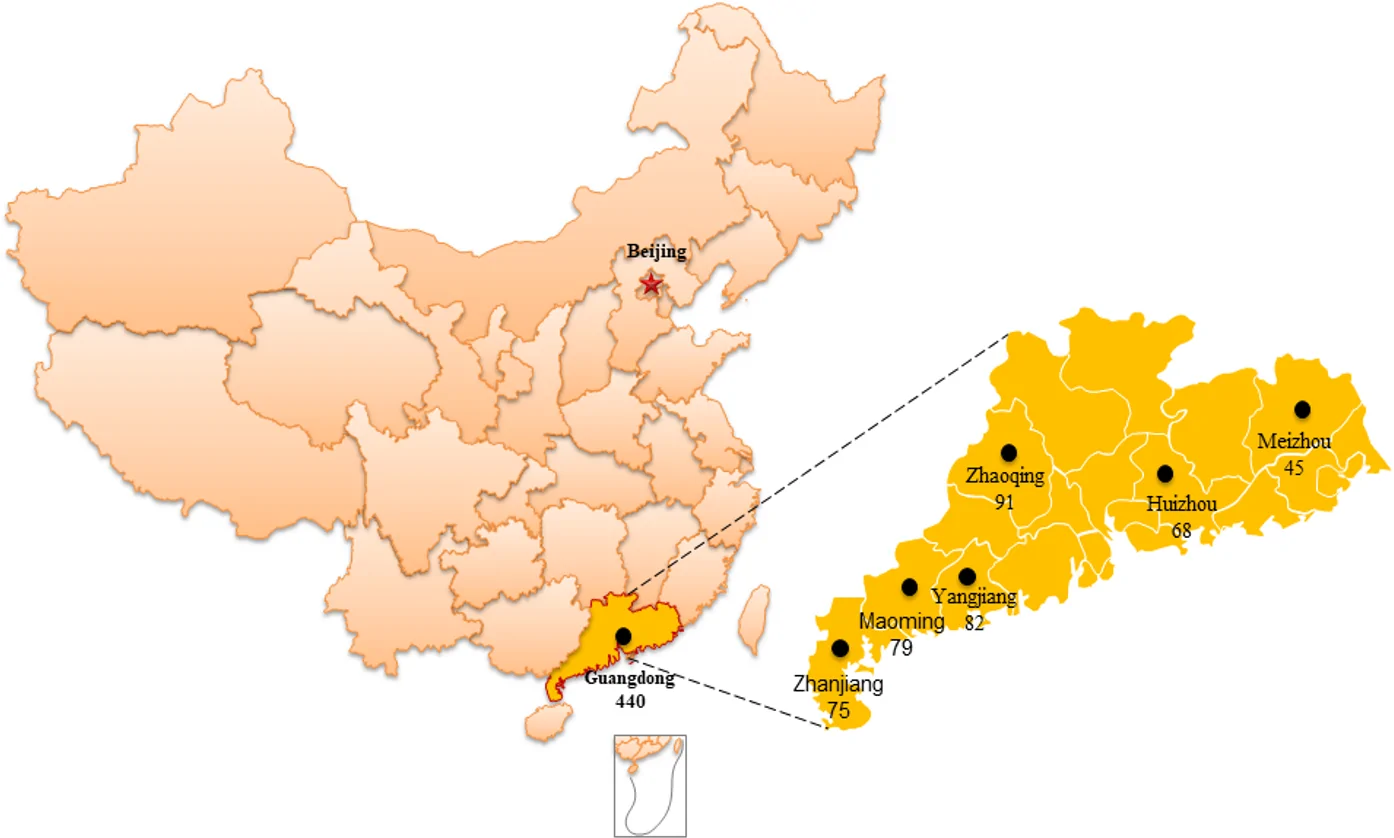
The geographical distribution of 440 porcine clinical samples was marked with black dots in Guangdong Province of China. The numbers listed in the figure indicated the total number of samples collected in six cities within Guangdong Province in 2023.

To investigate the prevalence of PTVs in Guangdong Province, China, a total of 341 fecal samples and 99 tissue mixture samples (including liver, spleen, stomach, brain, or intestinal tissues) were collected from pigs of various age groups across six cities (Maoming, Zhanjiang, Yangjiang, Zhaoqing, Meizhou, and Huizhou) in 2023. The detailed breakdown of sample numbers is presented in Fig. 1. Animals exhibiting clinical signs such as diarrhea, respiratory symptoms, or neurological signs were included. All sample processing adhered to standardized operating procedures.

### Viral RNA extraction and virus detection

Fecal samples were homogenized in 1 mL of ice-cold phosphate-buffered saline (PBS) supplemented with 1% penicillin/streptomycin (P/S). Similarly, tissue mixture samples were resuspended in 1 mL of ice-cold PBS containing 1% P/S and processed in a ball mill. The resulting homogenates were centrifuged at 12,000 × *g* for 20 min at 4°C using an Eppendorf 5418 R centrifuge (Eppendorf, Germany) to obtain a clear supernatant. From this supernatant, 0.2 mL was carefully transferred into a 2 mL Eppendorf tube. Viral RNA was then extracted using the UE Body Fluid Virus DNA/RNA Small Scale Preparation Kit (Suzhou Eyerbright, China), following the manufacturer’s instructions. Subsequently, the extracted viral RNA was reverse-transcribed into complementary DNA (cDNA) using the PrimeScript RT Master Mix (TaKaRa, Japan), adhering to the manufacturer’s recommended protocol.

The synthesized viral cDNA was subjected to conventional Polymerase Chain Reaction (PCR) using the specific primer pair (PF0-F/PF0-R, detailed in Table 1) and a PCR kit from Vazyme (China). The PCR amplification was performed under the following cycling conditions: an initial denaturation step at 94°C for 3 min; followed by 35 cycles, each consisting of denaturation at 94°C for 30 s, annealing at 54°C for 30 s, and extension at 72°C for 30 s; and culminating in a final extension step at 72°C for 7 min. The resulting PCR products were analyzed by agarose gel electrophoresis to confirm the presence and size of the target viral cDNA.

**TABLE 1:** Primers for amplifying the full-length genome of PTV/CN/GD/316

Primers	Oligonucleotide sequence (5′−3′)^*a*^	Position	Product size (bp)	References
PF0-F	TGTTGTGTTTAAACACAGAAAT	113–134	306	(25)
PF0-R	TTCAACTGACTATACAAAGTAC	398–419		
PF1-F	AGRGTACAGAAGAGCAAG	144–161	1,650	
PF1-R	TGCACRTAATCARAAAATTCWCCWGGCA	1767–1794		
PF2-F	ATACCCAARNTWGAYATTGATGC	1661–1683	1,873	
PF2-R	GGRTCWGTYTTNGCAAATTTCTT	3512–3534		
PF3-F	ATTTTGAAAGAYCCAGCRGTYGATGC	3343–3368	1,453	
PF3-R	TTTTCTTCAGAATTTTCYTTTGCTCTCA	4769–4796		
PF4-F	AAACARCAAGCYGTCACAATWATTGA	4139–4167	1,837	
PF4-R	TTCCACARGTCAGTCCGGCGCAAGCCYTTCTT	5945–5976		
PF5-F	TGGGGCATHCGWTGGGCCTCATA	5789–5811	1,229	
PF5-R	TTTTGCTCAACTAAAACTACT	6999–7018		

^*a*^ Degenerate base: R(A/G); W(A/T); Y(C/T); N(A/C/G/T); H(A/C/T).

### Virus isolation and genome sequencing

For fecal samples identified as PTV-positive by RT-PCR, the supernatants were collected and passed through sterile 0.22 µm filters. The filtered material, supplemented with 1% P/S, was then used to inoculate monolayers of swine testicular (ST) cells in six-well culture plates. Infection was initiated by incubating the cells with the filtered material for 2 h at 37°C in a 5% CO_2_ incubator. Following this adsorption period, fresh DMEM supplemented with 1% P/S was added, and the cultures were incubated for an additional 5 days. To facilitate virus release, cultures underwent two freeze-thaw cycles and were then centrifuged at 12,000 × *g* for 5 min at 4°C using an Eppendorf 5418 R centrifuge (Eppendorf, Germany). The clarified supernatants were subsequently used to inoculate fresh ST cells. This process was repeated for six serial passages, with culture supernatants collected at each passage and stored at −80°C until further use.

For PTV genome sequencing, cDNA was amplified using FastPfu Fly DNA polymerase (TransGen Biotech, China) and LA Taq (TaKaRa, Japan). Six pairs of overlapping PCR primers were designed to amplify the polyprotein gene of the PTV/CN/GD/316 strain. Primer design was based on published genomic sequences of PTV reference strains (Table 1). The resulting PCR products were purified by agarose gel electrophoresis and then subjected to sequencing.

### Growth kinetics

To determine the replication kinetics of the PTV/CN/GD/316 strain, ST cells were utilized. ST cell monolayers were cultured in six-well plates to approximately 80%–90% confluence and maintained at 37°C with 5% CO_2_. Cells were then infected with PTV at an MOI of 0.1. At 6 h intervals post-infection, cell supernatants were collected to perform 50% tissue culture infective dose (TCID_50_) assays. Viral titers were quantified using the Reed-Muench method. The resulting growth curves were plotted and analyzed using GraphPad Prism 8.0.

### Electron microscopy

Supernatants from PTV-infected ST cell cultures were centrifuged at 12,000 × *g* for 5 min at 4°C using an Eppendorf 5418 R centrifuge (Eppendorf, Germany) to remove the precipitate. Viral particles were then concentrated using a universal virus concentration kit (Beyotime, China) following the manufacturer’s protocol. The concentrated virus was resuspended in 500 µL of the supernatant and applied to a copper grid for negative staining with 3% phosphotungstic acid (pH 7.0). The stained samples were examined using electron microscopy.

### Detection of other swine pathogens and co-infection rate analysis

To comprehensively assess the co-infection landscape, PTV-positive samples were further screened for the presence of seven other common swine viruses: Porcine Epidemic Diarrhea Virus (PEDV), PCV2, Porcine Circovirus type 3 (PCV3), PRV, PRRSV, CSFV, and Porcine Deltacoronavirus (PDCoV). Viral DNA and RNA were concurrently extracted from all PTV-positive samples using the Axygen Body Fluid Viral DNA/RNA Kit (Corning, USA) following the manufacturer’s instructions. This RNA extraction also enabled subsequent reverse transcription for RNA virus detection.

Pathogen detection strategies were tailored to the viral genome type. For DNA viruses, namely PCV2, PCV3, and PRV, conventional PCR was employed. The amplification reactions utilized the 2× Taq Plus Master Mix (Vazyme, China), and primers specifically designed for these eight porcine viruses (Table 2). The conventional PCR thermal cycling protocol was identical to that described above. For RNA viruses, including PEDV, PRRSV, CSFV, and PDCoV, a one-step RT-PCR kit (Vazyme, China) was utilized. The RT-PCR amplification conditions were optimized as follows: an initial reverse transcription step at 50°C for 30 min; pre-denaturation at 94°C for 3 min; followed by 35 cycles of denaturation at 94°C for 15 s, gradient annealing at 55°C–72°C for 30 s, and extension at 72°C for 60 s, with a final extension at 72°C for 7 min. Reactions were then held at 4°C. The RT-PCR products were analyzed by agarose gel electrophoresis.

**TABLE 2:** Primers for the detection of swine viral pathogens

Primers	Sequences 5′−3′^*a*^	Product size (bp)	Purpose	References
PEDV S-F	GGTGTTAAGTTTACGTCCCT	468	PEDV detection	(26)
PEDV S-R	AAGTGGGACATAGCCAATAC			
PCV2-DF	CACGGATATTGTAGTCCTGG	500	PCV2 detection	
PCV2-DR	CCGCACCTTCGGATATACTGTC			
PCV3-DF	GATCCACGGAGGTCTGTAGG	357	PCV3 detection	(27)
PCV3-DR	CACGTACCCTTGCAAGTGTG			
PRV gE-F	ACGAGCCCCGCTTCCACGCG	316	PRV detection	(26)
PRV gE-R	CACCGGTCGCCGAGCAGCGG			
PRRSV ORF7-F	CCAGCCAGTCAATCARCTGTG	292	PRRSV detection	
PRRSV ORF7 -R	GCGAATCAGGCGCACWGTATG			
CSFV-F	GCAGAAGCCCACCTCGAGAT	245	CSFV detection	
CSFV-R	TACACCGGTTCCTCCACTC			
PDCoV M-F	ATCCTCCARGGAGGCTATGC	494	PDCoV detection	(28)
PDCoV M-R	GCRAATTCTGGATCGTTGTT			
PTV-VP1-F	CCNGCNGARACAGGHTGTG	816	PTV classification	(18)
PTV-VP1-R	TCYTCAACATCYCCKGCYT			

^*a*^ Degenerate Base: R(A/G); W(A/T); Y(C/T); N(A/C/G/T); H(A/C/T); K(G/T).

### Phylogenetic and genetic analyses

To elucidate the evolutionary relationships among the PTV isolates and established genotypes, phylogenetic analysis was conducted using complete genomic sequences and VP1 sequences. Multiple sequence alignments were performed separately for two data sets using the MUSCLE algorithm in MEGA v11. The first data set included the whole-genome sequence of the PTV/CN/GD/316 strain, aligned with 30 reference sequences representing 11 distinct genotypes (Table S2). The second data set comprised 21 newly amplified PTV VP1 sequences, compared with 56 VP1 reference sequences representing 18 distinct genotypes (Tables S1 and S3).

Maximum-likelihood (ML) phylogenetic trees were constructed using the optimal substitution model selected by the Bayesian Information Criterion (BIC). Branch support was evaluated through 1,000 bootstrap replicates, with values ≥ 70% displayed at branch nodes to indicate confidence in the most recent common ancestor of supported clades. The final phylogenetic trees were visually enhanced and annotated for clarity using the online tool ChiPlot (29).

## RESULTS

### Prevalence of PTVs in Guangdong Province, China

To assess the prevalence of PTVs in pigs, a comprehensive investigation was conducted in Guangdong Province. RNA was extracted from 440 samples and analyzed using specific primers via RT-PCR (Table 1). The overall positivity rate for PTVs was 19.32% (85 out of 440 clinical samples). Among these, the positivity rate was 17.17% (17 out of 99) in tissue mixture samples and 19.94% (68 out of 341) in fecal samples (Table 3).

**TABLE 3:** Positive rates of PTVs in different types of the samples in 2023^*a*^

Sample types	PTVs positive rate (%) (positive/total)
T-pool	17.17% (17/99)
Feces	19.94% (68/341)
Total	19.32% (85/440)

^*a*^ T-pool: the tissue mixture samples include liver, spleen, stomach, brain, or intestinal tissues.

### Virus isolation and identification

**Fig 2:**
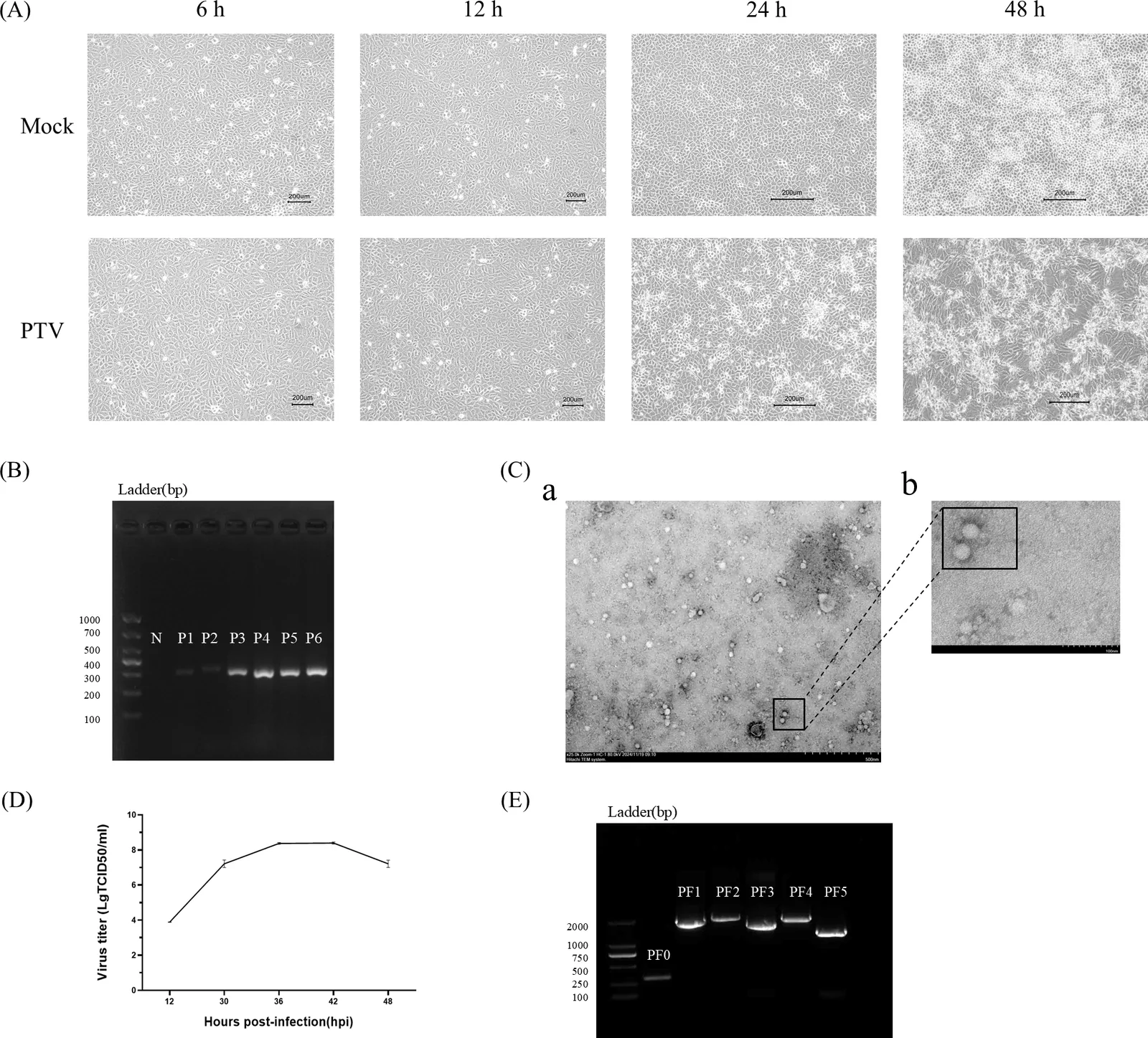
Isolation and characterization of PTV (PTV/CN/GD/316). (**A**) The cytopathic effect in ST cells with or without PTV inoculation (bar = 200 µm). (**B**) RT-PCR identification of the PTV/CN/GD/316 strain after ST cells infection. P1–P6: generation from 1 to 6 of the cell culture isolate; N: negative. (**C**) Electron microscopy of particles of the PTV/CN/GD/316 strain (a: bar = 500 nm; b: bar = 100 nm). (**D**) One-step growth curve of the PTV/CN/GD/316 strain. (**E**) Gel electrophoresis analysis of whole-genome RT-PCR amplification products of PTV. PF0-PF5: the products of RT-PCR amplification using the primers from Table 1.

PTV was successfully isolated from a tissue mixture sample that tested positive for PTV, PRRSV, and PEDV, but negative for PCV2, PCV3, PRV, CSFV, and PDCoV. After six blind passages, only PTV was detected, with no evidence of PRRSV or PEDV. Following the sixth blind passage, approximately 60% of the cells exhibited cytopathic effects (CPE), including shrinkage, rounding, and detachment, while the control group showed no such changes (Fig. 2A).

Viral RNA was extracted from each generation of the viral culture, and RT-PCR was performed using the primers PF0-F/PF0-R (Table 1). As shown in Fig. 2B, viral cultures were successfully amplified from the 1st to the 6th passage, with product sizes consistent with the expected size of 306 bp. After concentrating the cell culture supernatants, virus particles with diameters of approximately 25–30 nm were observed under an electron microscope, consistent with small RNA viruses (Fig. 2C).

The growth kinetics of PTV in ST cells demonstrated rapid replication, reaching a peak titer of approximately 10^8.37^ TCID_50_/mL at 36 h post-infection, followed by a gradual decline (Fig. 2D). These results confirm the successful isolation of a PTV strain, designated PTV/CN/GD/316.

### Genome sequencing

To further characterize the genomic information of the PTV/CN/GD/316 strain, six pairs of overlapping primers were used to amplify the full-length genome directly from cell culture, followed by Sanger sequencing (Fig. 2E). The complete genome of the PTV/CN/GD/316 strain was determined to be 6,999 nucleotides in length and has been deposited in the GenBank database under the accession number PV165534.1.

### Co-infection with other porcine pathogens

PTVs are frequently associated with co-infection involving other porcine pathogens. To investigate this, we screened 85 PTV-positive samples for the presence of common porcine viruses, including PEDV, PRV, PCV2, PRRSV, CSFV, PCV3, and PDCoV (Table 4). Among the PTV-positive samples, co-infection with PCV2, PEDV, PRRSV, PCV3, PDCoV, and CSFV were observed in 96.47% (82/85), 21.18% (18/85), 18.82% (16/85), 8.24% (7/85), 3.53% (3/85), and 1.18% (1/85) of cases, respectively. Notably, no PRV was detected in any of the PTV-positive samples. The co-infection rate with PCV2 was significantly higher compared to other viruses. Additionally, instances of triple, quadruple, and even quintuple infections involving these viruses were observed (Table 4). Overall, 97.65% (83/85) of the PTV-positive samples exhibited co-infection with at least one other virus. These findings align with previous studies, confirming that PTV, PCV2, PEDV, and PRRSV have a high prevalence in Chinese pig herds and demonstrating that co-infection with PTV and other viruses is a common occurrence (20–23).

**TABLE 4:** Co-infection rate of PTV-positive samples with other common swine viruses (*n* = 85)

Co-infection status	Co-infection status in 85 PTV-positive sample			
	Virus	Positive number	Percentage	Total percentage
Dual infections	PEDV	1	1.18%	61.18%
	PCV2	51	60.00%	
Triple infections	PCV2 + PEDV	9	10.58%	23.53%
	PCV2 + PRRSV	8	9.41%	
	PCV2 + PCV3	2	2.35%	
	PCV2 + PDCoV	1	1.18%	
Quadruple infections	PCV2 + PEDV + PRRSV	4	4.70%	10.59%
	PCV2 + PEDV + PCV3	2	2.35%	
	PCV2 + PEDV + PDCoV	1	1.18%	
	PCV2 + PRRSV + PCV3	1	1.18%	
	PCV2 + PRRSV + PDCoV	1	1.18%	
Quintuple infections	PCV2 + PRRSV + CSFV + PCV3	1	1.18%	2.35%
	PCV2 + PEDV + PRRSV + PCV3	1	1.18%	
Total	PEDV	18	21.18%	97.65%^*a*^
	PRV	0	0.00%	
	PCV2	82	96.47%	
	PRRSV	16	18.82%	
	CSFV	1	1.18%	
	PCV3	7	8.24%	
	PDCoV	3	3.53%	

^*a*^ The results showed that 83 of 85 (97.65%) PTV-positive samples were co-infected with at least one virus.

### Phylogenetic and genetic analysis of PTV

**Fig 3:**
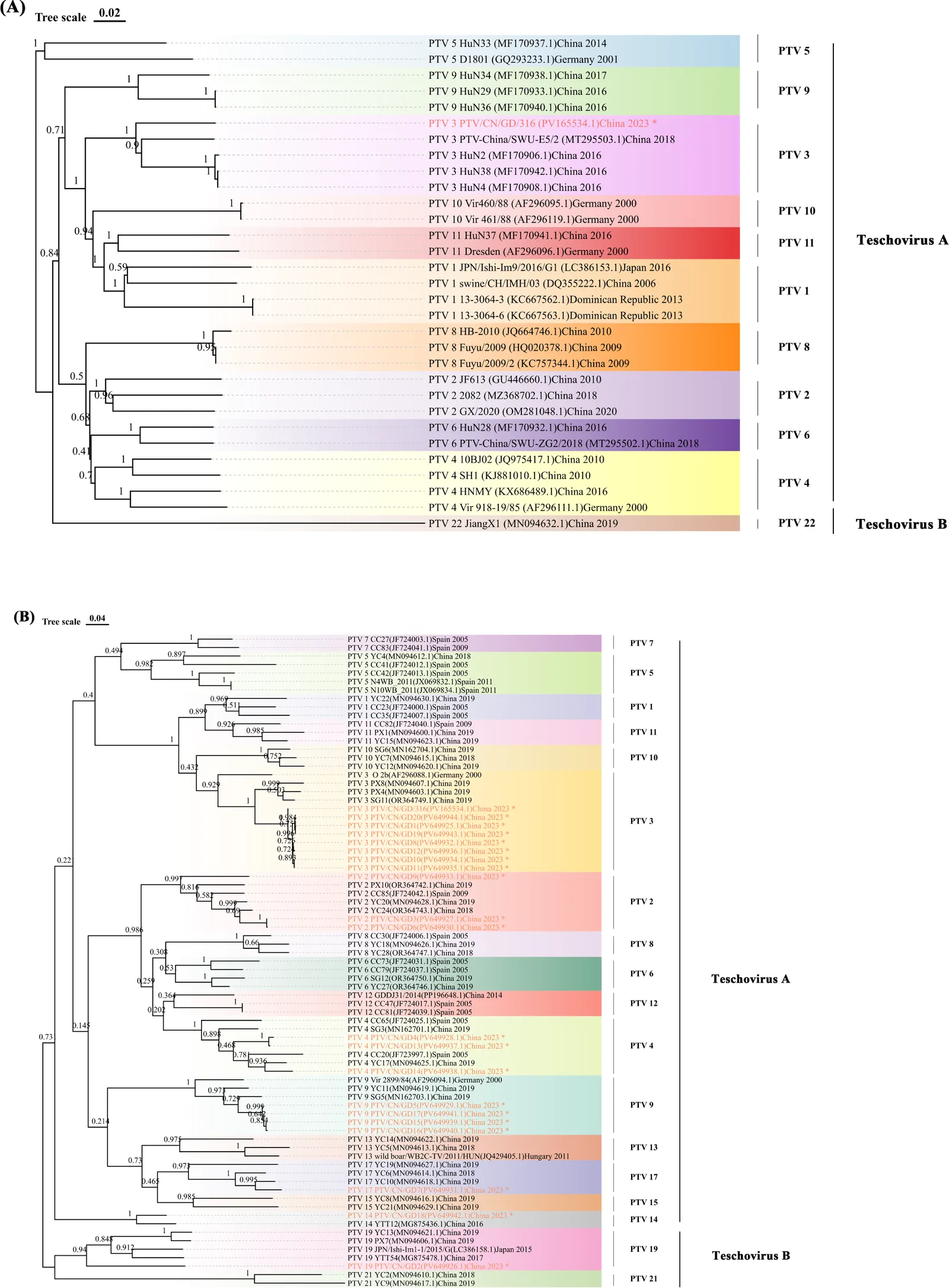
Phylogenetic analysis of PTV strains. Phylogenetic trees were constructed using the ML method with 1,000 bootstrap replicates based on the BIC in MEGA v11, and were subsequently modified using ChiPlot for visual enhancement. (**A**) Phylogenetic analysis and sequence alignment of PTV/CN/GD/316 with 30 reference strains based on the complete genomic sequences of PTVs. (**B**) Phylogenetic analysis and sequence alignment of the 21 obtained PTV strains with 56 reference strains based on VP1 sequences of PTVs. The strains obtained in this study are marked with an asterisk (*).

The VP1 protein, being the major component of the viral capsid and the most diversified viral protein, is commonly used for phylogenetic analysis to characterize PTV genotypes. To elucidate the genetic relationships of the PTV/CN/GD/316 strain, the Maximum-likelihood (ML) phylogenetic trees were constructed using 1,000 bootstrap replicates, based on the VP1 sequence and the full genome sequence of the strain. The results revealed that PTV/CN/GD/316 clustered closely with PTV genotype 3 and exhibited a strong genetic relationship with HuN2, HuN38, and HuN4 (Fig. 3).

In this study, the VP1 coding regions of 21 PTV strains, including PTV/CN/GD/316, were obtained from 85 PTV-positive samples through RT-PCR amplification. These strains were named PTV/CN/GD1 to PTV/CN/GD20 and deposited in the GenBank database under accession numbers PV649925 to PV649944 (Table S1). According to the phylogenetic tree of the complete VP1 genes from the 21 isolated PTV strains, the result showed that three strains belong to PTV2 (14.29%), eight strains belong to PTV3 (38.10%), three strains belong to PTV4 (14.29%), four strains belong to PTV9 (19.05%), one strain belongs to PTV14 (4.76%), one strain belongs to PTV17 (4.76%), and one strain belongs to PTV19 (4.76%) (Fig. 3B).

## DISCUSSION

PTV was first detected in Europe, has become enzootic in Europe and other regions, establishing themselves as one of the major infectious pathogens affecting swine worldwide (8). Recent studies have reported its widespread prevalence in several Chinese regions, including Shanghai, Hunan, Shandong, Hebei, Heilongjiang, Jilin, and Tianjin (18, 21, 25, 30, 31). However, the prevalence of PTVs in Guangdong Province remained unexplored prior to this study. Our investigation revealed a PTV positivity rate of 19.32% in swine herds in Guangdong Province, which is notably lower than rates reported in other regions of China, such as Shanghai (85.7%) and Northeast China (61.3%) (18, 21). Key variables include geographical location, rearing conditions, and the health status of swine herds. Additionally, differences in sample types, sample sizes, and immune responses within the studied populations could influence the observed prevalence. Some studies have reported higher PTV infection rates in asymptomatic pigs compared to those showing clinical signs such as diarrhea (18, 25). In contrast, our study exclusively analyzed clinical samples from pigs exhibiting neurological, diarrheal, or respiratory symptoms, which may explain the relatively lower PTV positivity rate observed here. The potential for asymptomatically infected pigs to act as reservoirs and vectors underscores a major limitation of symptom-based surveillance. Relying solely on clinical presentation may lead to substantial underestimation of regional pathogen prevalence and compromise the effectiveness of biosecurity measures. To address this gap and prevent unforeseen outbreaks, we strongly recommend incorporating asymptomatic herds into future routine surveillance programs.

PTVs are associated with a diverse range of clinical symptoms, including encephalomyelitis, diarrhea, reproductive disorders, and respiratory symptoms. This clinical variability is likely linked to the genetic and serotypic diversity of PTVs (32, 33). The VP1 protein, one of the four capsid proteins of PTVs, is the most diversified and is commonly used as a reliable marker for genotyping and serotyping PTV strains (5). Therefore, we performed phylogenetic tree analysis and sequence alignment on the 21 obtained PTV strains with 56 reference strains from the GenBank based on the VP1 gene. The results identified seven distinct genotypes among the 21 PTV strains: PTV2, PTV3, PTV4, PTV9, PTV14, PTV17, and PTV19. Notably, PTV3 was the most prevalent genotype (38.10%), followed by PTV9 (19.05%), PTV2 (14.29%), PTV4 (14.29%), PTV14 (4.76%), PTV17 (4.76%), and PTV19 (4.76%). This observation contrasts with findings from previous studies in Shanghai (18) and Hunan (25), where PTV4 was reported as the most prevalent genotype. Such regional differences in genotype distribution underscore the complexity and dynamic nature of PTV epidemiology. Since the first documented PTV strain (Swine/CH/IMH/03) in China’s Inner Mongolia Region in 2003, numerous epidemiological studies have been conducted across the country (19). To date, 23 PTV genotypes have been reported globally, with 21 of these identified in China. The only genotypes not yet reported in China are PTV7 and PTV23 (17–19, 21, 25, 30, 31). These findings highlight the highly diversified and uneven distribution of PTV genotypes, both within China and globally.

Previous studies have reported the persistent survival of PTVs in pig farms, which increases the likelihood of co-infection with other common swine pathogens, such as PRRSV, CSFV, PRV, PCV2, PEDV, and TGEV, in various regions of China (20, 23). These co-infections not only amplify their pathogenic potential but also pose significant challenges for disease control and prevention. Therefore, continuous surveillance of PTVs is imperative to better understand their epidemiological dynamics and co-infection patterns with other pathogens. Accordingly, we also investigated the co-infection status of PTVs in Guangdong Province. The results revealed that PTVs are frequently found in co-infection with PEDV, PRV, PCV2, PRRSV, PCV3, CSFV, and PDCoV, with dual, triple, or quadruple infections commonly observed. The highest co-infection rate was noted with PCV2, which can be attributed to the high prevalence of PCV2 in China. This high prevalence of PCV2 can be explained by several factors: (i) the genetic diversity and high mutation rate of PCV2 undermine the protective efficacy of vaccines; (ii) intensive farming practices and suboptimal biosecurity measures on some farms promote its horizontal transmission; and (iii) the immunosuppressive state induced by PCV2 in the host, primarily marked by lymphopenia, depletion of lymphoid cells (B cells and T cells), inhibition of interferon production, and so on, ultimately creates a permissive environment for other pathogens (34, 35). These factors may account for the high co-infection rate of PTVs with PCV2. Additionally, PEDV and PDCoV are currently the predominant pathogens causing diarrhea in Chinese swine populations (36, 37). Consequently, a high co-infection rate with PEDV was detected in PTV-positive diarrheal fecal samples, while the co-infection rate with PDCoV was relatively low in this study. Interestingly, PRV and CSFV were rarely detected in both fecal and tissue samples. This likely reflects the success of control measures in some areas of Guangdong Province, China, such as CSFV vaccination and PRRSV eradication programs, which have significantly reduced infection rates and viral shedding. These findings underscore the importance of sustained vaccination and disease management strategies in controlling the spread of these pathogens.

### Conclusions

This investigation provides compelling evidence of the widespread dissemination of PTVs in Guangdong Province, China. Phylogenetic analysis identified PTV3 as the predominant genotype circulating in the region in 2023, followed by PTV9. These findings enhance our understanding of the genetic evolution of PTV strains in China and offer valuable insights for future research on these viral pathogens. Furthermore, the frequent occurrence of co-infection between PTVs and other porcine pathogens highlights the potential for exacerbated clinical symptoms and substantial economic losses for livestock farms. In summary, this study contributes critical information regarding the prevalence and evolutionary dynamics of PTVs, which will inform the development of effective prevention and control strategies. By deepening our knowledge of PTV epidemiology and its interactions during co-infection with pathogens, we can better safeguard swine health and mitigate the economic impact of these infections on the agricultural industry.

## Supplementary Material

Reviewer comments

## Data Availability

All data from this experiment are available from the corresponding author upon request.

## SUPPLEMENTAL MATERIAL

Tables S1 to S3 spectrum.04214-25-s0001.docx The information of PTV strains obtained in this study.

OPEN PEER REVIEW reviewer-comments.pdf An accounting of the reviewer comments and feedback.

ASM does not own the copyrights to Supplemental Material that may be linked to, or accessed through, an article. The authors have granted ASM a non-exclusive, world-wide license to publish the Supplemental Material files. Please contact the corresponding author directly for reuse.
