# Supplementary material for: The prevalence and molecular characterization of Porcine teschoviruses in Guangdong Province, China
Source: Microbiol Spectr. 2026 Sep 9;14(10):e04214-25. doi: 10.1128/spectrum.04214-25 (PMC13689215; doi:10.1128/spectrum.04214-25)
Supplement: Supplementary file 1 [file reviewer-comments.pdf]

AMERICAN SOCIETY FOR MICROBIOLOGY | Microbiology Spectrum

The Prevalence and Molecular Characterization of Porcine Teschoviruses (PTVs) in Guangdong Province, China

Xingyao Feng, Zirui Tang, Mingjie Liu, Shengnan Chen, Feng Wen, Kaiyin Hu, Lisha Chen, Rui Zhou, and Jinyue Guo

Corresponding Author(s): Jinyue Guo, Foshan University School of Life Science and Engineering

Review Timeline:

Submission Date:	January 3, 2026
Editorial Decision:	February 12, 2026
Revision Received:	April 1, 2026
Accepted:	May 5, 2026

Editor: Michael Becker

Reviewer(s): The reviewers have opted to remain anonymous.

Transaction Report:

(Note: With the exception of the correction of typographical or spelling errors that could be a source of ambiguity, letters and reports are not edited. The original formatting of letters and referee reports may not be reflected in this compilation.)

DOI: https://doi.org/10.1128/spectrum.04214-25

Re: Spectrum04214-25 (The Prevalence and Molecular Characterization of Porcine Teschoviruses (PTVs) in Guangdong Province, China)

Dear Dr. Jinyue Guo:

Thank you for the privilege of reviewing your work. Below you will find my comments, instructions from the Spectrum editorial office, and the reviewer comments.

Both reviewers were generally positive about the manuscript, noting its scientific value, sound methodology, and overall clarity. However, they have suggested several revisions to improve clarity, consistency, and contextual discussion of the findings. I agree that these revisions will strengthen the manuscript. Please carefully address all reviewer comments in a point-by-point response and revise the manuscript accordingly.

Please return the manuscript within 60 days; if you cannot complete the modification within this time period, please contact me. If you do not wish to modify the manuscript and prefer to submit it to another journal, notify me immediately so that the manuscript may be formally withdrawn from consideration by Spectrum.

Revision Guidelines
To submit your modified manuscript, log into the submission site at https://spectrum.msubmit.net/cgi-bin/main.plex. Go to Author Tasks and click the appropriate manuscript title to begin. The information you entered when you first submitted the paper will be displayed; update this as necessary. Note the following requirements:

• Upload point-by-point responses to the issues raised by the reviewers in a file named "Response to Reviewers," NOT in your cover letter.
• Upload a compare copy of the manuscript (without figures) as a "Marked-Up Manuscript" file.
• Upload a clean .DOC/.DOCX version of the revised manuscript and remove the previous version.
• Each figure must be uploaded as a separate, editable, high-resolution file (TIFF or EPS preferred), and any multipanel figures must be assembled into one file.
• Any supplemental material intended for posting by ASM should be uploaded with their legends separate from the main manuscript. You can combine all supplemental material into one file (preferred) or split it into a maximum of 10 files with all associated legends included.

For complete guidelines on revision requirements, see our Submission and Review Process webpage. Submission of a paper that does not conform to guidelines may delay acceptance of your manuscript.

Data availability: ASM policy requires that data be available to the public upon online posting of the article, so please verify all links to sequence records, if present, and make sure that each number retrieves the full record of the data. If a new accession number is not linked or a link is broken, provide Spectrum production staff with the correct URL for the record. If the accession numbers for new data are not publicly accessible before the expected online posting of the article, publication may be delayed; please contact production staff (Spectrum@asmusa.org) immediately with the expected release date.

Publication Fees: For information on publication fees and which article types are subject to charges, visit our website. If your manuscript is accepted for publication and any fees apply, you will be contacted separately about payment during the production process; please follow the instructions in that e-mail. Arrangements for payment must be made before your article is published.

ASM Membership: Corresponding authors may join or renew ASM membership to obtain discounts on publication fees. Need to upgrade your membership level? Please contact Customer Service at Service@asmusa.org.

The ASM Journals program strives for constant improvement in our submission and publication process. Please tell us how we can improve your experience by taking this quick Author Survey.

Thank you for submitting your paper to Spectrum.

Sincerely,
Michael Becker
Editor
Microbiology Spectrum

Reviewer #1 (Comments for the Author):

This manuscript presents a valuable and comprehensive study on the prevalence, molecular characterization, and co-infection

dynamics of Porcine Teschoviruses (PTVs) in Guangdong Province, China. The methodology is sound, involving a large sample size, successful virus isolation, full genome sequencing of a novel strain, and detailed phylogenetic and co-infection analyses. The findings, particularly concerning the high co-infection rates and the specific genotype distribution in this region, are important for swine health management. The manuscript is generally well-written and clearly structured. However, a few minor revisions are necessary to improve clarity, consistency, and the presentation of data, particularly regarding the genotypic classification and the presentation of co-infection data. I recommend minor revisions to address the concerns outlined below.

1.Inconsistency Check: Please confirm which genotype is truly the most prevalent based on the VP1 analysis (Figure 3B). If PTV 3 (35%) is the highest, then the statement "followed by PTV 9" needs to be verified against the actual percentages (PTV 9 is 20%, PTV 2 is 15%, PTV 4 is 15%). Please ensure the ranking in the Abstract and Discussion accurately reflects the proportions derived from the VP1 analysis.
2.The presentation of Table 4 is slightly confusing because the columns mix different aggregation levels (e.g., dual infections vs. individual virus prevalence). The text correctly summarizes the overall co-infection rate (97.65%). I recommend explicitly stating in the text or a caption note how the 97.65% overall co-infection rate was calculated (i.e., 85 total positive samples minus the samples positive for PTV only).
3.(L340): The statement contrasts the prevalence here with Shanghai (85.7%) and Northeast China (61.3%). It might be beneficial to mention why those rates were higher (e.g., different sample types, different genotyping methods, or simply regional differences) to reinforce the impact of the current study's findings.
4.The high co-infection rate with PCV2 (96.47%) is highlighted, but the biological implications (e.g., immunosuppression synergy) are superficially discussed. Expand on mechanistic hypotheses.
5.Materials and Methods:
Sample collection: Clarify the "tissue mixture samples" composition (line 116). What tissues were pooled?
Ethics statement: Missing details on animal ethical approval; include if applicable.
Electron microscopy: The caption for Figure 2C mentions "bar= 500 nm or 100 nm"-specify which scale applies to which panel.
6.Tables 1-4 are useful, but add footnotes to explain abbreviations (e.g., "R" in primers).

Reviewer #2 (Comments for the Author):

This manuscript reports a comprehensive epidemiological study on Porcine Teschoviruses (PTVs) in Guangdong Province, China. While the work contributes meaningfully to the understanding of PTV circulation and diversity in swine populations, several substantive and presentational issues require attention prior to publication. Specifically, the following points merit careful revision:

1. The study relies exclusively on clinical samples from pigs exhibiting overt clinical signs. This sampling strategy may lead to an underestimation of the true regional prevalence of PTV infection. The authors are requested to explicitly acknowledge this limitation and discuss its implications for the reported overall prevalence rate of 19.32%. Furthermore, recommendations for future surveillance, including systematic sampling of asymptomatic herds, should be incorporated into the Discussion section.
2. The classification of strain "PTV/CN/GD/316" as a novel PTV-3 genotype requires rigorous justification aligned with current International Committee on Taxonomy of Viruses (ICTV) criteria for genotype demarcation. Please clarify whether this isolate represents a newly defined genotype or a distinct strain within the established PTV-3 clade, supported by phylogenetic and pairwise nucleotide divergence analyses.
3. The exceptionally high co-infection rate (97.65%), particularly with Porcine Circovirus Type 2 (PCV2), warrants more nuanced interpretation. In addition to general considerations of immunosuppression, the authors should propose context-specific hypotheses, grounded in local husbandry practices, co-circulating pathogens, or environmental factors, to explain the observed epidemiological association in Guangdong Province.
4. The legend for Figure 2E erroneously cites Table 2. It should instead reference Table 1, which lists the primers used for PTV genome amplification. This correction must be implemented consistently across the manuscript and figure captions.
5. Terminological consistency must be ensured throughout the manuscript. Key terms, including but not limited to "prevalence," "genotype," "strain," "co-infection," and "serotype", should be defined upon first use and applied uniformly thereafter. A terminology checklist is recommended during final proofreading.
6. Given that serial passage of the isolated PTV strain was performed in cell culture and considering the well-documented propensity of RNA viruses to accumulate adaptive mutations during in vitro propagation, the authors are asked to confirm whether VP1 gene sequencing was conducted across multiple passages. Such data are essential to verify genetic stability and ensure accurate genotypic assignment.
7. Due to the inherent challenges in attributing clinical manifestations to individual agents in polymicrobial infections, functional validation of the isolated PTV strain is rerecommended. Specifically, controlled animal challenge experiments in pathogen-free pigs would provide critical evidence regarding its in vivo pathogenicity and clinical relevance.

This manuscript presents a valuable and comprehensive study on the prevalence, molecular characterization, and co-infection dynamics of Porcine Teschoviruses (PTVs) in Guangdong Province, China. The methodology is sound, involving a large sample size, successful virus isolation, full genome sequencing of a novel strain, and detailed phylogenetic and co-infection analyses. The findings, particularly concerning the high co-infection rates and the specific genotype distribution in this region, are important for swine health management. The manuscript is generally well-written and clearly structured. However, a few minor revisions are necessary to improve clarity, consistency, and the presentation of data, particularly regarding the genotypic classification and the presentation of co-infection data. I recommend minor revisions to address the concerns outlined below.

1. Inconsistency Check: Please confirm which genotype is truly the most prevalent based on the VP1 analysis (Figure 3B). If PTV 3 (35%) is the highest, then the statement “followed by PTV 9” needs to be verified against the actual percentages (PTV 9 is 20%, PTV 2 is 15%, PTV 4 is 15%). Please ensure the ranking in the Abstract and Discussion accurately reflects the proportions derived from the VP1 analysis.
2. The presentation of Table 4 is slightly confusing because the columns mix different aggregation levels (e.g., dual infections vs. individual virus prevalence). The text correctly summarizes the overall co-infection rate (97.65%). I recommend explicitly stating in the text or a caption note how the 97.65% overall co-infection rate was calculated (i.e., 85 total positive samples minus the samples positive for PTV only).
3. (L340): The statement contrasts the prevalence here with Shanghai (85.7%) and Northeast China (61.3%). It might be beneficial to mention *why* those rates were higher (e.g., different sample types, different genotyping methods, or simply regional differences) to reinforce the impact of the current study’s findings.
4. The high co-infection rate with PCV2 (96.47%) is highlighted, but the biological implications (e.g., immunosuppression synergy) are superficially discussed. Expand on mechanistic hypotheses.
5. Materials and Methods:
- Sample collection: Clarify the "tissue mixture samples" composition (line 116). What tissues were pooled?
- Ethics statement: Missing details on animal ethical approval; include if applicable.
- Electron microscopy: The caption for Figure 2C mentions "bar= 500 nm or 100 nm"—specify which scale applies to which panel.

6. Tables 1–4 are useful, but add footnotes to explain abbreviations (e.g., "R" in primers).

This manuscript reports a comprehensive epidemiological study on Porcine Teschoviruses (PTVs) in Guangdong Province, China. While the work contributes meaningfully to the understanding of PTV circulation and diversity in swine populations, several substantive and presentational issues require attention prior to publication. Specifically, the following points merit careful revision:

1. The study relies exclusively on clinical samples from pigs exhibiting overt clinical signs. This sampling strategy may lead to an underestimation of the true regional prevalence of PTV infection. The authors are requested to explicitly acknowledge this limitation and discuss its implications for the reported overall prevalence rate of 19.32%. Furthermore, recommendations for future surveillance, including systematic sampling of asymptomatic herds, should be incorporated into the Discussion section.
2. The classification of strain "PTV/CN/GD/316" as a novel PTV-3 genotype requires rigorous justification aligned with current International Committee on Taxonomy of Viruses (ICTV) criteria for genotype demarcation. Please clarify whether this isolate represents a newly defined genotype or a distinct strain within the established PTV-3 clade, supported by phylogenetic and pairwise nucleotide divergence analyses.
3. The exceptionally high co-infection rate (97.65%), particularly with Porcine Circovirus Type 2 (PCV2), warrants more nuanced interpretation. In addition to general considerations of immunosuppression, the authors should propose context-specific hypotheses, grounded in local husbandry practices, co-circulating pathogens, or environmental factors, to explain the observed epidemiological association in Guangdong Province.
4. The legend for Figure 2E erroneously cites Table 2. It should instead reference Table 1, which lists the primers used for PTV genome amplification. This correction must be implemented consistently across the manuscript and figure captions.
5. Terminological consistency must be ensured throughout the manuscript. Key terms, including but not limited to "prevalence," "genotype," "strain," "co-infection," and "serotype", should be defined upon first use and applied uniformly thereafter. A terminology checklist is recommended during final proofreading.

6. Given that serial passage of the isolated PTV strain was performed in cell culture and considering the well-documented propensity of RNA viruses to accumulate adaptive mutations during in vitro propagation, the authors are asked to confirm whether VP1 gene sequencing was conducted across multiple passages. Such data are essential to verify genetic stability and ensure accurate genotypic assignment.

7. Due to the inherent challenges in attributing clinical manifestations to individual agents in polymicrobial infections, functional validation of the isolated PTV strain is rerecommended. Specifically, controlled animal challenge experiments in pathogen-free pigs would provide critical evidence regarding its *in vivo* pathogenicity and clinical relevance.

Dear Editors and Reviewers

Thank you for your letter and for the reviewers' The Prevalence and Molecular Characterization of Porcine Teschoviruses (PTVs) in Guangdong Province, China (Spectrum04214-25). Those comments are valuable and very helpful for revising and improving our paper, as well as the important guiding significance to our researches. We have studied the comments carefully and have made corrections which we hope meet with approval. Revised portions are marked with tracking changes in the revised paper. The main corrections in the paper and the responds to the reviewer's comments are as flowing:

Editor and Reviewer Comments:

Reviewer #1

1: Inconsistency Check: Please confirm which genotype is truly the most prevalent based on the VP1 analysis (Figure 3B). If PTV 3 (35%) is the highest, then the statement "followed by PTV 9" needs to be verified against the actual percentages (PTV 9 is 20%, PTV 2 is 15%, PTV 4 is 15%). Please ensure the ranking in the Abstract and Discussion accurately reflects the proportions derived from the VP1 analysis.

Response: We thank the reviewer for highlighting this inconsistency. Based on the phylogenetic analysis of the complete VP1 gene, PTV 3 was indeed the most prevalent genotype (38.10%), followed by PTV 9 (19.05%), PTV 2 (14.29%), PTV 4 (14.29%), PTV 14 (4.76%), PTV 17 (4.76%), and PTV 19 (4.76%). The corresponding descriptions in the Abstract (lines 43–44) and Discussion (lines 345–347) have been revised to accurately reflect these percentages and the resulting genotype ranking. Meanwhile, The percentages for each genotype here have been adjusted, primarily to include the PTV/CN/GD/316 strain, which was not included in the original manuscript.

2: The presentation of Table 4 is slightly confusing because the columns mix different aggregation levels (e.g., dual infections vs. individual virus prevalence). The text correctly summarizes the overall co-infection rate (97.65%). I recommend explicitly stating in the text or a caption note how the 97.65% overall co-infection rate was calculated (i.e., 85 total positive samples minus the samples positive for PTV only).

Response: Thank you for your valuable feedback and suggestions on our manuscript. A footnote has also been added to explicitly explain the calculation of the overall co-infection rate.

3: L340): The statement contrasts the prevalence here with Shanghai (85.7%) and

Northeast China (61.3%). It might be beneficial to mention why those rates were higher (e.g., different sample types, different genotyping methods, or simply regional differences) to reinforce the impact of the current study's findings.

Response: Thank you very much for your comment. Our investigation revealed a PTV positivity rate of 19.32% in swine herds in Guangdong, which is notably lower than rates reported in other regions of China, such as Shanghai (85.7%) and Northeast China (61.3%)[18, 21]. Several factors may contribute to these regional discrepancies in PTV prevalence. Geographical location, rearing conditions, and the health status of swine herds are among the primary variables influencing infection rates. Additionally, differences in sample types, sample sizes, and immune responses within the studied populations could affect the observed prevalence. We have added the reasons for the discrepancies in the results to the discussion section (lines 324-331).

4: The high co-infection rate with PCV2 (96.47%) is highlighted, but the biological implications (e.g., immunosuppression synergy) are superficially discussed. Expand on mechanistic hypotheses.

Response: Thank you for your valuable feedback and suggestions. In the Discussion section, we further elucidated the reasons why PCV2-induced immunosuppression leads to a high co-infection rate with PTV. It can be attributed to the high prevalence of PCV2 in China. This high prevalence of PCV2 can be explained by several factors: (i) The genetic diversity and high mutation rate of PCV2 undermine the protective efficacy of vaccines; (ii) Intensive farming practices and suboptimal biosecurity measures on some farms promote its horizontal transmission; (iii) The immunosuppressive state induced by PCV2 in the host, primarily marked by lymphopenia, depletion of lymphoid cells (B cells and T cells), inhibition of interferon production and so on, ultimately creates a permissive environment for other pathogens[34-35]. These factors may account for the high co-infection rate of PTVs with PCV2. We have added these contents to the discussion section (lines 368-376).

5: Materials and Methods:
Sample collection: Clarify the "tissue mixture samples" composition (line 116). What tissues were pooled?
Ethics statement: Missing details on animal ethical approval; include if applicable.
Electron microscopy: The caption for Figure 2C mentions "bar= 500 nm or 100 nm"- specify which scale applies to which panel.

Response: Thank you for your valuable feedback and suggestions. (1) We have revised the manuscript to specify that the tissue mixture samples include liver, spleen, stomach, brain, or intestinal tissues (lines 117-118). (2) Ethics statement has been added to materials and methods section (lines 226-230). (3) The scale bar for the electron

microscopy image in Figure 2C has been further detailed in the figure legend (a: bar = 500 nm; b: bar = 100 nm) (lines 259-260).

6: Tables 1-4 are useful, but add footnotes to explain abbreviations (e.g., "R" in primers).

Response: Thank you very much for your comment. R: A/G; W: A/T; N: A/C/G/T;Y: C/T ;H: A/C/T. We have added footnotes to explain the abbreviations in Tables 1-4.

Reviewer #2

1: The study relies exclusively on clinical samples from pigs exhibiting overt clinical signs. This sampling strategy may lead to an underestimation of the true regional prevalence of PTV infection. The authors are requested to explicitly acknowledge this limitation and discuss its implications for the reported overall prevalence rate of 19.32%. Furthermore, recommendations for future surveillance, including systematic sampling of asymptomatic herds, should be incorporated into the Discussion section.

Response: Thank you for your valuable feedback and suggestions. Some studies on experimentally infected PTV case have reported encephalomyelitis, diarrhea, reproductive disorders, and respiratory symptoms. However, PTV infections are most often asymptomatic. Yang et al. and Sun et al.found that the infection rate of PTV in asymptomatic pigs was significantly higher than that in pigs with diarrhea. However, our study exclusively analyzed clinical samples from pigs with neurological, diarrheal, or respiratory symptoms, which may explain the relatively lower PTV positivity rate than that reported by Yang et al. and Sun et al. The potential for asymptomatically infected pigs to act as reservoirs and vectors for pathogen spread underscores a critical limitation of symptom-based surveillance. Reliance solely on clinical presentation may lead to a significant underestimation of regional pathogen prevalence and compromise biosecurity efficacy. To address this gap and prevent unforeseen outbreaks, the integration of asymptomatic herds into future routine surveillance programs is strongly recommended. We have added your suggestions to the Discussion section (lines 331-337).

2: The classification of strain "PTV/CN/GD/316" as a novel PTV-3 genotype requires rigorous justification aligned with current International Committee on Taxonomy of Viruses (ICTV) criteria for genotype demarcation. Please clarify whether this isolate represents a newly defined genotype or a distinct strain within the established PTV-3 clade, supported by phylogenetic and pairwise nucleotide divergence analyses.

Response: Thank you very much for your comment. According to phylogenetic and pairwise nucleotide divergence analyses, PTV/CN/GD/316 strain represents a distinct

strain within the established PTV 3 clade. We have revised the description of the PTV/CN/GD/316 strain in the manuscript accordingly.

3: The exceptionally high co-infection rate (97.65%), particularly with Porcine Circovirus Type 2 (PCV2), warrants more nuanced interpretation. In addition to general considerations of immunosuppression, the authors should propose context-specific hypotheses, grounded in local husbandry practices, co-circulating pathogens, or environmental factors, to explain the observed epidemiological association in Guangdong Province.

Response: Thank you for your valuable feedback and suggestions. The highest co-infection rate was noted with PCV2, which can be attributed to the high prevalence of PCV2 in China. This high prevalence of PCV2 can be explained by several factors: (i) The genetic diversity and high mutation rate of PCV2 undermine the protective efficacy of vaccines; (ii) Intensive farming practices and suboptimal biosecurity measures on some farms promote its horizontal transmission; (iii) The immunosuppressive state induced by PCV2 in the host, primarily marked by lymphopenia, depletion of lymphoid cells (B cells and T cells), inhibition of interferon production and so on, ultimately creates a permissive environment for other pathogens[34-35]. These factors may account for the high co-infection rate of PTVs with PCV2. We have added the contents to the Discussion section (lines 372-380).

4: The legend for Figure 2E erroneously cites Table 2. It should instead reference Table 1, which lists the primers used for PTV genome amplification. This correction must be implemented consistently across the manuscript and figure captions.

Response: Thank you for your suggestions. We have corrected Table 2 to Table 1 in the Figure 2E legend and ensured consistency across the manuscript and figure captions.

5: Terminological consistency must be ensured throughout the manuscript. Key terms, including but not limited to "prevalence," "genotype," "strain," "co-infection," and "serotype", should be defined upon first use and applied uniformly thereafter. A terminology checklist is recommended during final proofreading.

Response:: Thank you for your valuable feedback and suggestions. We have revised the terminology throughout the manuscript to ensure consistency.

6: Given that serial passage of the isolated PTV strain was performed in cell culture and considering the well-documented propensity of RNA viruses to accumulate adaptive mutations during in vitro propagation, the authors are asked to confirm whether VP1 gene sequencing was conducted across multiple passages. Such data are essential to verify genetic stability and ensure accurate genotypic assignment.

Response: Thank you for your insightful comment. VP1 gene sequencing and genotypic analysis were performed not only on the early-passage virus (P6) used in the manuscript, but also on the original primary isolate and the virus at passage 13. Comparative analysis revealed a high degree of conservation, with greater than 99% nucleotide identity among these passages. The minimal sequence variations observed are most likely attributable to technical artifacts introduced during sequencing or PCR amplification, rather than representing biologically significant adaptive mutations.

7: Due to the inherent challenges in attributing clinical manifestations to individual agents in polymicrobial infections, functional validation of the isolated PTV strain is recommended. Specifically, controlled animal challenge experiments in pathogen-free pigs would provide critical evidence regarding its in vivo pathogenicity and clinical relevance.

Response: Thank you for this insightful comment. We fully agree that in polymicrobial infections, functional validation is essential to establish the causal relationship between a specific pathogen and the observed clinical manifestations. We acknowledge that controlled challenge experiments in pathogen-free pigs would provide definitive evidence of the pathogenicity of the isolated PTV strain. However, as the primary objective of this study is to provide a comprehensive molecular epidemiological and genomic baseline for this novel PTV strain, we have structured our research plan to address these findings systematically. We have already initiated preparations for controlled animal challenge studies as an independent follow-up investigation, which will specifically focus on the clinical relevance and in vivo pathogenicity of this strain. We believe this two-tiered approach establishing genomic data first, followed by experimental validation will provide the most robust framework for understanding the clinical significance of this pathogen.

Re: Spectrum04214-25R1 (The Prevalence and Molecular Characterization of Porcine Teschoviruses (PTVs) in Guangdong Province, China)

Dear Dr. Jinyue Guo:

Thank you for your revised manuscript, all concerns of the peer reviewers have been addressed. Your manuscript has been accepted, and I am forwarding it to the ASM production staff for publication. Your paper will first be checked to make sure all elements meet the technical requirements. ASM staff will contact you if anything needs to be revised before copyediting and production can begin. Otherwise, you will be notified when your proofs are ready to be viewed.

Data Availability: ASM policy requires that data be available to the public upon online posting of the article, so please verify all links to sequence records, if present, and make sure that each number retrieves the full record of the data. If a new accession number is not linked or a link is broken, provide production staff with the correct URL for the record. If the accession numbers for new data are not publicly accessible before the expected online posting of the article, publication may be delayed; please contact ASM production staff immediately with the expected release date.

Publication Fees: For information on publication fees and which article types have charges, please visit our website. We have partnered with Copyright Clearance Center (CCC) to collect author charges. If fees apply to your paper, you will receive a message from no-reply@copyright.com with further instructions. For questions related to paying charges through RightsLink, please contact CCC at ASM_Support@copyright.com or toll free at +1-877-622-5543. CCC makes every attempt to respond to all emails within 24 hours.

ASM Membership: Corresponding authors may join or renew ASM membership to obtain discounts on publication fees. Need to upgrade your membership level? Please contact Customer Service at Service@asmusa.org.

Embargo Policy: A press release may be issued as soon as the manuscript is posted on the Spectrum Latest Articles webpage. The corresponding author will receive an email with the subject line "ASM Journals Author Services Notification" when the article is available online.

The ASM Journals program strives for constant improvement in our submission and publication process. Please tell us how we can improve your experience by taking this quick Author Survey.

Thank you for submitting your paper to Spectrum.

Sincerely,
Michael Becker
Editor
Microbiology Spectrum

Reviewer #1 (Comments for the Author):

The revisions and responses provided by the authors have addressed the concerns raised in this manuscript review.

Reviewer #2 (Comments for the Author):

All of my concerns have been adequately addressed.
