# Supplementary material for: The prevalence and molecular characterization of Porcine teschoviruses in Guangdong Province, China
Source: Microbiol Spectr. 2026 Sep 9;14(10):e04214-25. doi: 10.1128/spectrum.04214-25 (PMC13689215; doi:10.1128/spectrum.04214-25)
Supplement: Supplementary file 2 [file spectrum.04214-25-s0001.docx]

**Table S1.** The information of PTV strains obtained in this study.

| No. | Accession Number | Strains | Country | Year | Host | Length | Genotype |
| --- | --- | --- | --- | --- | --- | --- | --- |
| 1 | PV649925.1 | PTV/CN/GD1 | China | 2023 | pig | 762 bp | PTV 3 |
| 2 | PV649926.1 | PTV/CN/GD2 | China | 2023 | pig | 768 bp | PTV 19 |
| 3 | PV649927.1 | PTV/CN/GD3 | China | 2023 | pig | 768 bp | PTV 2 |
| 4 | PV649928.1 | PTV/CN/GD4 | China | 2023 | pig | 765 bp | PTV 4 |
| 5 | PV649929.1 | PTV/CN/GD5 | China | 2023 | pig | 759 bp | PTV 9 |
| 6 | PV649930.1 | PTV/CN/GD6 | China | 2023 | pig | 768 bp | PTV 2 |
| 7 | PV649931.1 | PTV/CN/GD7 | China | 2023 | pig | 768 bp | PTV 17 |
| 8 | PV649932.1 | PTV/CN/GD8 | China | 2023 | pig | 762 bp | PTV 3 |
| 9 | PV649933.1 | PTV/CN/GD9 | China | 2023 | pig | 768 bp | PTV 2 |
| 10 | PV649934.1 | PTV/CN/GD10 | China | 2023 | pig | 762 bp | PTV 3 |
| 11 | PV649935.1 | PTV/CN/GD11 | China | 2023 | pig | 762 bp | PTV 3 |
| 12 | PV649936.1 | PTV/CN/GD12 | China | 2023 | pig | 762 bp | PTV 3 |
| 13 | PV649937.1 | PTV/CN/GD13 | China | 2023 | pig | 765 bp | PTV 4 |
| 14 | PV649938.1 | PTV/CN/GD14 | China | 2023 | pig | 765 bp | PTV 4 |
| 15 | PV649939.1 | PTV/CN/GD15 | China | 2023 | pig | 759 bp | PTV 9 |
| 16 | PV649940.1 | PTV/CN/GD16 | China | 2023 | pig | 759 bp | PTV 9 |
| 17 | PV649941.1 | PTV/CN/GD17 | China | 2023 | pig | 759 bp | PTV 9 |
| 18 | PV649942.1 | PTV/CN/GD18 | China | 2023 | pig | 759 bp | PTV 14 |
| 19 | PV649943.1 | PTV/CN/GD19 | China | 2023 | pig | 762 bp | PTV 3 |
| 20 | PV649944.1 | PTV/CN/GD20 | China | 2023 | pig | 762 bp | PTV 3 |
| 21 | PV165534.1 | PTV/CN/GD316 | China | 2023 | pig | 6999 bp | PTV 3 |

**Table S2.** The information of 30 referenced complete genomic sequences of PTVs.

| No. | Accession Number | Strains | Country | Year | Host | Length | Genotype |
| --- | --- | --- | --- | --- | --- | --- | --- |
| 1 | MF170937.1 | HuN33 | China | 2014 | Swine | 6796 bp | PTV 5 |
| 2 | GQ293233.1 | D1801 | Germany | 2001 | Pig | 7117 bp | PTV 5 |
| 3 | MF170938.1 | HuN34 | China | 2017 | Swine | 6793 bp | PTV 9 |
| 4 | MF170933.1 | HuN29 | China | 2016 | Swine | 6840 bp | PTV 9 |
| 5 | MF170940.1 | HuN36 | China | 2016 | Swine | 6790 bp | PTV 9 |
| 6 | MT295503.1 | PTV-China/SWU-E5/2 | China | 2018 | Tibetan pig | 6760 bp | PTV 3 |
| 7 | MF170906.1 | HuN2 | China | 2016 | Swine | 6850 bp | PTV 3 |
| 8 | MF170942.1 | HuN38 | China | 2016 | Swine | 6862 bp | PTV 3 |
| 9 | MF170908.1 | HuN4 | China | 2016 | Swine | 6868 bp | PTV 3 |
| 10 | AF296095.1 | Vir460/88 | Germany | 2000 | - | 7009 bp | PTV 10 |
| 11 | AF296119.1 | Vir 461/88 | Germany | 2000 | - | 7009 bp | PTV 10 |
| 12 | MF170941.1 | HuN37 | China | 2016 | Swine | 6856 bp | PTV 11 |
| 13 | AF296096.1 | Dresden | Germany | 2000 | Dresden Pig | 7111 bp | PTV 11 |
| 14 | LC386153.1 | JPN/Ishi-Im9/2016/G1 | Japan | 2016 | Sus scrofa | 7096 bp | PTV 1 |
| 15 | DQ355222.1 | swine/CH/IMH/03 | China | 2006 | - | 6860 bp | PTV 1 |
| 16 | KC667563.1 | 13-3064-6 | Dominican Republic | 2013 | Swine | 7155 bp | PTV 1 |
| 17 | KC667562.1 | 13-3064-3 | Dominican Republic | 2013 | Swine | 7155 bp | PTV 1 |
| 18 | JQ664746.1 | HB-2010 | China | 2010 | Swine | 7090 bp | PTV 8 |
| 19 | HQ020378.1 | Fuyu/2009 | China | 2009 | Swine | 7066 bp | PTV 8 |
| 20 | KC757344.1 | Fuyu/2009/2 | China | 2009 | Pig | 7128 bp | PTV 8 |
| 21 | GU446660.1 | JF613 | China | 2010 | - | 7051 bp | PTV 2 |
| 22 | MZ368702.1 | 2082 | China | 2018 | Swine | 7019 bp | PTV 2 |
| 23 | OM281048.1 | GX/2020 | China | 2020 | Pigs | 7015 bp | PTV 2 |
| 24 | MF170932.1 | HuN28 | China | 2016 | Swine | 6845 bp | PTV 6 |
| 25 | MT295502.1 | PTV-China/SWU-ZG2/2018 | China | 2018 | Tibetan pig | 6766 bp | PTV 6 |
| 26 | JQ975417.1 | 10BJ02 | China | 2010 | Pig | 6972 bp | PTV 4 |
| 27 | KJ881010.1 | SH1 | China | 2010 | Porcine | 7097 bp | PTV 4 |
| 28 | KX686489.1 | HNMY | China | 2016 | Pig | 7192 bp | PTV 4 |
| 29 | AF296111.1 | Vir 918-19/85 | Germany | 2000 | - | 7015 bp | PTV 4 |
| 30 | MN094632.1 | JiangX1 | China | 2019 | Swine | 6799 bp | PTV 22 |

**Table S3.** The information of 56 referenced VP1 sequences of PTV.

| No. | Accession Number | Strains | Country | Year | Host | Length | Genotype |
| --- | --- | --- | --- | --- | --- | --- | --- |
| 1 | JF724003.1 | CC27 | Spain | 2005 | Swine | 786 bp | PTV 7 |
| 2 | JF724041.1 | CC83 | Spain | 2009 | Swine | 786 bp | PTV 7 |
| 3 | MN094612.1 | YC4 | China | 2018 | Swine | 783 bp | PTV 5 |
| 4 | JF724012.1 | CC41 | Spain | 2005 | Swine | 786 bp | PTV 5 |
| 5 | JF724013.1 | CC42 | Spain | 2005 | Swine | 783 bp | PTV 5 |
| 6 | JX069832.1 | N4WB_2011 | Spain | 2011 | Sus scrofa (wild boar) | 783 bp | PTV 5 |
| 7 | JX069834.1 | N10WB_2011 | Spain | 2011 | Sus scrofa (wild boar) | 783 bp | PTV 5 |
| 8 | MN094630.1 | YC22 | China | 2019 | Swine | 786 bp | PTV 1 |
| 9 | JF724000.1 | CC23 | Spain | 2005 | Swine | 786 bp | PTV 1 |
| 10 | JF724007.1 | CC35 | Spain | 2005 | Swine | 786 bp | PTV 1 |
| 11 | JF724040.1 | CC82 | Spain | 2009 | Swine | 789 bp | PTV 11 |
| 12 | MN094600.1 | PX1 | China | 2019 | Swine | 789 bp | PTV 11 |
| 13 | MN094623.1 | YC15 | China | 2019 | Swine | 789 bp | PTV 11 |
| 14 | MN162704.1 | SG6 | China | 2019 | Swine | 786 bp | PTV 10 |
| 15 | MN094615.1 | YC7 | China | 2018 | Swine | 786 bp | PTV 10 |
| 16 | MN094620.1 | YC12 | China | 2019 | Swine | 786 bp | PTV 10 |
| 17 | AF296088.1 | O 2b | Germany | 2000 | - | 7012 bp | PTV 3 |
| 18 | MN094607.1 | PX8 | China | 2019 | Swine | 786 bp | PTV 3 |
| 19 | MN094603.1 | PX4 | China | 2019 | Swine | 786 bp | PTV 3 |
| 20 | OR364749.1 | SG11 | China | 2019 | Swine | 786 bp | PTV 3 |
| 21 | OR364742.1 | PX10 | China | 2019 | Swine | 792 bp | PTV 2 |
| 22 | JF724042.1 | CC85 | Spain | 2009 | Swine | 792 bp | PTV 2 |
| 23 | MN094628.1 | YC20 | China | 2019 | Swine | 792 bp | PTV 2 |
| 24 | OR364743.1 | YC24 | China | 2018 | Swine | 792 bp | PTV 2 |
| 25 | JF724006.1 | CC30 | Spain | 2005 | Swine | 792 bp | PTV 8 |
| 26 | MN094626.1 | YC18 | China | 2019 | Swine | 792 bp | PTV 8 |
| 27 | OR364747.1 | YC28 | China | 2018 | Swine | 792 bp | PTV 8 |
| 28 | JF724031.1 | CC73 | Spain | 2005 | Swine | 792 bp | PTV 6 |
| 29 | JF724037.1 | CC79 | Spain | 2005 | Swine | 792 bp | PTV 6 |
| 30 | OR364750.1 | SG12 | China | 2019 | Swine | 792 bp | PTV 6 |
| 31 | OR364746.1 | YC27 | China | 2019 | Swine | 792 bp | PTV 6 |
| 32 | PP196648.1 | GDDJ31/2014 | China | 2014 | Swine | 792 bp | PTV 12 |
| 33 | JF724017.1 | CC47 | Spain | 2005 | Swine | 792 bp | PTV 12 |
| 34 | JF724039.1 | CC81 | Spain | 2005 | Swine | 792 bp | PTV 12 |
| 35 | JF724025.1 | CC65 | Spain | 2005 | Swine | 789 bp | PTV 4 |
| 36 | MN162701.1 | SG3 | China | 2019 | Swine | 789 bp | PTV 4 |
| 37 | JF723997.1 | CC20 | Spain | 2005 | Swine | 789 bp | PTV 4 |
| 38 | MN094625.1 | YC17 | China | 2019 | Swine | 789 bp | PTV 4 |
| 39 | AF296094.1 | Vir 2899/84 | Germany | 2000 | - | 7006 bp | PTV 9 |
| 40 | MN094619.1 | YC11 | China | 2019 | Swine | 783 bp | PTV 9 |
| 41 | MN162703.1 | SG5 | China | 2019 | Swine | 783 bp | PTV 9 |
| 42 | MN094622.1 | YC14 | China | 2019 | Swine | 786 bp | PTV 13 |
| 43 | MN094613.1 | YC5 | China | 2019 | Swine | 786 bp | PTV 13 |
| 44 | JQ429405.1 | Wild boar/WB2C-TV/2011/HUN | Hungary | 2011 | Sus scrofa (wild boar) | 7123 bp | PTV 13 |
| 45 | MN094627.1 | YC19 | China | 2019 | Swine | 783 bp | PTV 17 |
| 46 | MN094614.1 | YC6 | China | 2018 | Swine | 786 bp | PTV 17 |
| 47 | MN094618.1 | YC10 | China | 2019 | Swine | 786 bp | PTV 17 |
| 48 | MN094616.1 | YC8 | China | 2019 | Swine | 786 bp | PTV 15 |
| 49 | MN094629.1 | YC21 | China | 2019 | Swine | 786 bp | PTV 15 |
| 50 | MG875436.1 | YTT12 | China | 2016 | Swine | 786 bp | PTV 14 |
| 51 | MN094621.1 | YC13 | China | 2019 | Swine | 792 bp | PTV 19 |
| 52 | MN094606.1 | PX7 | China | 2019 | Swine | 792 bp | PTV 19 |
| 53 | LC386158.1 | JPN/Ishi-Im1-1/2015/G | Japan | 2015 | Sus scrofa | 6820 bp | PTV 19 |
| 54 | MG875478.1 | YTT54 | China | 2017 | Swine | 792 bp | PTV 19 |
| 55 | MN094610.1 | YC2 | China | 2018 | Swine | 792 bp | PTV 21 |
| 56 | MN094617.1 | YC9 | China | 2019 | Swine | 792 bp | PTV 21 |
